# A Forgotten Cause of Allergy at ER That Is Still Difficult to Diagnose and Treat at Poor Resource Setting: Angioedema after Using Angiotensin Converting Enzyme Inhibitors for 4 Years

**DOI:** 10.1155/2019/1676391

**Published:** 2019-01-02

**Authors:** A. A. Nilanga Nishad, K. Arulmoly, S. A. S. Priyankara, P. K. Abeysundara

**Affiliations:** ^1^Teaching Hospital, Batticaloa, Sri Lanka; ^2^North Colombo Teaching Hospital, Ragama, Sri Lanka

## Abstract

Angiotensin converting enzyme inhibitors (ACEi) are the most commonly used antihypertensives. Therefore, ACEI induced angioedema (ACEi-AE) is not uncommon. Physicians tend to miss the diagnosis whenever a patient is taking the drug for years due to misbelief of “a drug that was taken for years may not be the cause for an allergic reaction or an angioedema”. But ACEi can induce angioedema after many years of usage as well as sometimes after stopping the drug even. Most of the emergency physicians and centers are not aware of clinical diagnosis and diagnostic criteria including available diagnostic tests and more importantly the treatment options of ACEi-AE. Therefore not only the diagnosis is delayed or missing but also proper treatment options are not practiced at many emergency rooms and at wards.

## 1. Introduction

Angioedema is a life-threatening emergency due to the vast number of causes. It may be presented with or without pruritus and can be classified into allergic and nonallergic forms. The list of the drugs responsible for angioedema is on expansion and physician should be vigilant to find out its cause. Angiotensin converting enzyme inhibitors (ACEis) are very popular and mostly preferred antihypertensives used for different indications but known to cause angioedema. ACEi-associated angioedema results from decreased degradation of kinins and other vasoactive peptides such as substance P, thus cannot be treated with conventional antihistamines and steroids. More importantly angioedema can develop after using ACEi for a longer period of time and even after stopping it for sometime where medical personnel can be misled due to the fact that ACEi cannot be the cause as it has been used for a long time (Figures 2 and 3) [1]. We report a case of repeated angioedema episodes after using ACEis for 4 years.

## 2. Case Report

Sixty-eight-year old farmer was transferred to Teaching Hospital Batticaloa from a local hospital due to “allergic reaction”. On further questioning, patient is complaining of swelling of the face including the lips and difficulty in breathing with hoarseness of voices but without swallowing difficulty.

He had neither skin rashes nor pruritus. Other aspects of his medical history did not show any abnormality and were not significant as to the likely cause of his disease state. The patient could not come up with a possible food or contact history. He also had developed similar kind of attack two months back and was treated with antihistamines and steroids that time. He was having hypertension and was on Enalapril five milligrams two times daily for four years. Physical examination revealed a middle-aged man with swollen lips and lower part of the face. The pharynx was also oedematous. He was dyspnoeic. The breath sounds were vesicular and there were bilateral rhonchi. The pulse rate was 88 beats per minute and respiratory rate was 24 breaths per minute, respectively. His blood pressure was 110/70 mmHg. All other body system examination was essentially normal. A clinical assessment of Enalapril induced angioedema was made on clinical suspicion and Enalapril was immediately discontinued. He was treated with intramuscular Adrenaline 0.5 mg stat and intravenous hydrocortisone 200 mg stat and monitored for respiratory compromise. He was followed up with oral prednisolone 30 mg daily. We did not use C1-INH or Icatibant as it is not available in Sri Lanka. Patient did not need to undergo further management procedures. He was seen at the outpatient unit two days and one week later having recovered fully. The Naranjo probability scale indicated that this adverse drug event was probable [2].

## 3. Discussion

Angioedema is a life-threatening condition with swelling of the skin, mucosa, and submucosal tissues commonly seen on lips, tongue, face, hands, or feet [3]. Patients develop stridor and difficulty in breathing when it occurs in pharynx and larynx and rarely does it involve gastrointestinal tract and genitalia. It can be easily categorized as histamine-mediated or non-histamine-mediated. It can be presented with or without urticaria. Histamine-mediated angioedema usually presents with urticaria and swelling of the body that subsides within one to two days. It is also called allergic angioedema, an Ig-E-mediated hypersensitivity reaction with prior sensitization.

Bradykinin (Bk) is an inflammatory vasoactive peptide that leads to increased capillary permeability acting as a potent vasodilator. Bk-mediated angioedema comprises three distinct types: hereditary angioedema (HAE), acquired angioedema, and angiotensin-converting enzyme inhibitor (ACEi) induced angioedema [4]. Hereditary angioedema (C1-INH-HAE) results from increased bradykinin production and on the other hand ACEis block the effects of the angiotensin-converting enzyme, which impacts the renin-angiotensin-aldosterone pathway and diminishes the degradation of Bk. Therefore, ACEi-associated angioedema has been found to have resulted from decreased degradation of Bradykinin [5]. It usually occurs without urticaria. Recurrent angioedema is due to acquired C1-inhibitor deficiency (C1-INH-AAE).

**Figure 1:**
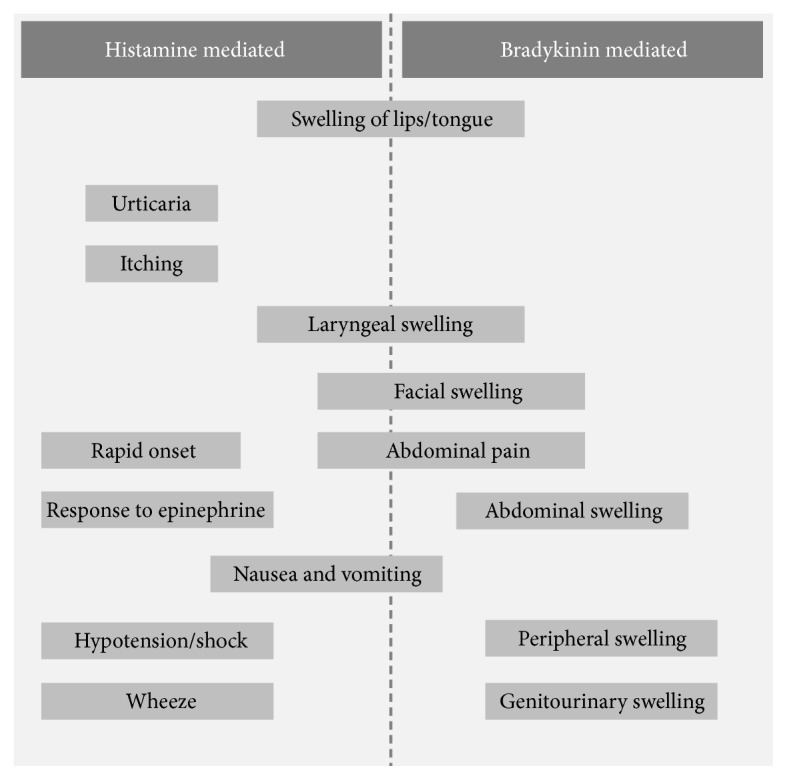
Distinguishing histamine-mediated versus bradykinin-mediated angioedema clinically (figure reproduced from Bernstein JA et al. (2017), [under the Creative Commons Attribution License/public domain]).

ACEi-AE are usually localized to the face or upper airway and upper portion of the digestive tract; they are importantly not characterized by erythema, but by swelling disorders, sometimes with urticaria followed by spontaneous remission (Figure 1) [7, 8]. Some other medications including nonsteroidal anti-inflammatory drugs, proton pump inhibitors, selective serotonin reuptake inhibitor, and other antidepressant can also produce this kind of angioedema [9].

ACEi-induced angioedema has been reported as a side effect affecting 0.1–0.7% of patients and up to 1.6% in some studies. Surprisingly 30 to 73% of angioedema cases recorded in emergency rooms are caused by ACEis [10]. A study carried out in United States reported an incidence of 0.2% nearly two decades ago [11]. Higher incidence has been reported in black patients, females, smokers, elderly, and those with a history of cough associated with ACEi use [12, 13]. The newest reviews suggested ACEi-induced angioedema prevalence to be ranging from 0.4 to 2.6 per 10,000 populations [14].

**Figure 2:**
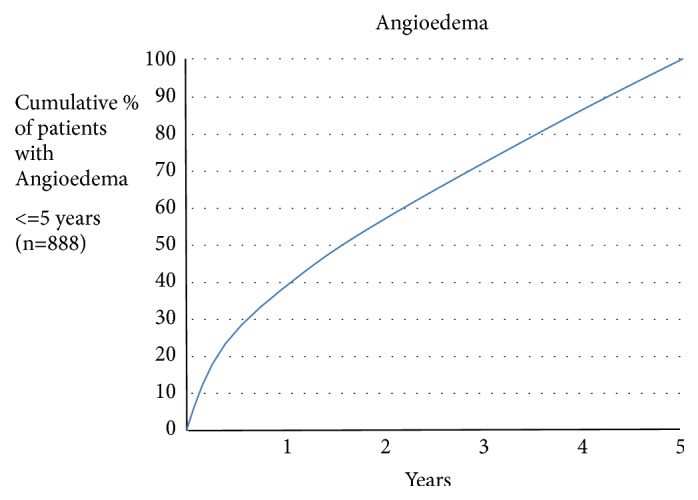
Cumulative percentages of developing angioedema and other side effects of ACEI with time (Banerji et al., 2017) [6].

**Figure 3:**
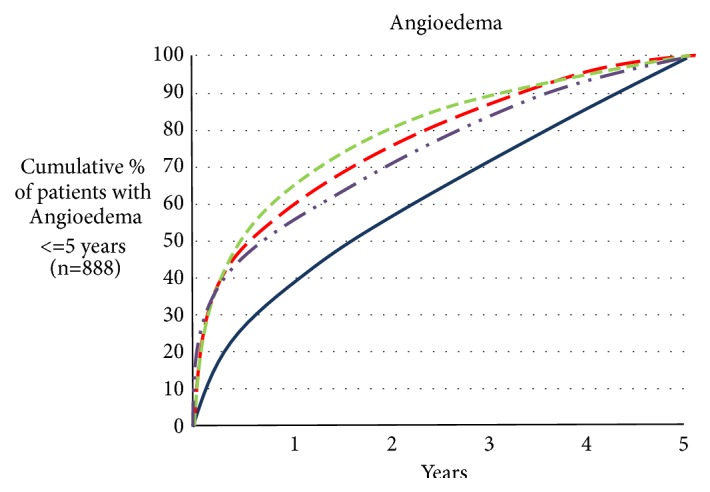
Cumulative percentages of developing angioedema and other side effects of ACEI with time (Banerji et al., 2017) [6].

Even though angioedema is the second most adverse event of ACEi next to dry cough, it is missed a lot due to healthcare provider believing that it should come just after starting the probable antigen (ACEi). Actually it is not the case. There are so many studies and case reports reporting that angioedema can develop at any time after starting the treatment with ACEis (Figures 2 and 3) [15]. A study following 134,945 patients for five years showed that, among the patients who developed ACEi-AE, nearly 20 percent developed it between 4th and 5th year [6]. Angioedema occurs in clusters until ACEi is stopped and it can occur even after stopping the treatment as a relapse sometimes up to 6 months of discontinuation (Figures 2 and 3) [16].

We have to rely more on clinical diagnosis (Figure 1) where most of the centers would not have enough facilities to diagnose the condition with plasma biochemical investigations (Table 1) [17].

Bradykinin-mediated angioedema is theoretically not responding to conventional treatment with glucocorticosteroids and antihistamines. Therefore, people have been treated with plasma derived C1-INH (C1 esterase inhibitor), recombinant C1-INH, Ecallantide, and Icatibant [18–21]. Unfortunately most of these treatment modalities are not available in our setup. Patients may rarely need to undergo endotracheal intubation and subsequent tracheostomy if the response is delayed and our patient does not need them.

## Figures and Tables

**Table 1:** Diagnostic tests to help distinguish among angioedema types (table reproduced from Bernstein JA et al. (2017), [under the Creative Commons Attribution License/public domain]).

**Type of angioedema**	**C1-INH concentration**	**C1-INH function**	**C4 concentration**	**Tryptase concentration** ^**∗**^
HAE type I	Low	Low	Low	Normal
HAE type II	Normal or High	Low	Low	Normal
HAE with normal C1-INH	Normal	Normal	Normal	Normal
Acquired AE	Low	Low	Low	Normal
ACEi-induced AE	Normal	Normal	Normal	Normal
Histamine-mediated anaphylaxis	Normal	Normal	Normal	Normal or Elevated

^**∗**^In blood drawn within 4–6 h of onset of attack.

*ACEi*: angiotensin-converting enzyme inhibitor; *AE*: angioedema; *C1-INH*: C1 inhibitor; *HAE*: hereditary angioedema.
